# L-Carnitine Improves the Asthma Control in Children with Moderate Persistent Asthma

**DOI:** 10.1155/2012/509730

**Published:** 2011-11-23

**Authors:** Mohammed Al-Biltagi, Mona Isa, Adel Salah Bediwy, Nevien Helaly, Dalia D. El Lebedy

**Affiliations:** ^1^Department of Paediatric, Faculty of Medicine, Tanta University, Tanta, P.o. box 1084, Gharbia Governate, Egypt; ^2^Department of Paediatric, Abu Alrish Pediatric Hospital, Faculty of Medicine, Cairo University, Cairo, Egypt; ^3^Department of Chest Diseases, Faculty of Medicine, Tanta University, Tanta, Gharbia Governate, Egypt; ^4^Department of Clinical and Chemical Pathology, Medical Division, National Research Center, Cairo, Egypt

## Abstract

*The objective*. was to investigate L-Carnitine level and the effects of its supplementation in children with moderate persistent Asthma. *Methods*. Free and total serum carnitine levels were measured in 50 children having moderate persistent asthma and 50 healthy control children. The patients group was randomly divided into two subgroups. Subgroup A was supplemented with L-carnitine for 6 months while subgroup B was used as a placebo controls. Both subgroups were assessed by pulmonary function tests (PFT) and childhood-asthma control test (C-ACT) before and 6 months after carnitine supplementation. *Results*. Total and free carnitine levels were significantly lower in patient group than in control group. PFT and C-ACT showed significant improvements in asthmatic children supplemented with L-carnitine than in those who were not supplemented. *Conclusion*. L-carnitine levels were initially lower in moderate persistent asthmatic children as compared to healthy control children. Asthmatic children who received L-carnitine supplementation showed statistically significant improvement of C-ACT and PFT.

## 1. Introduction

Asthma is a common complex chronic inflammatory disorder of the airways characterized by variable and recurring symptoms, airflow obstruction, bronchial hyper responsiveness, and an underlying inflammation. It is one of the most common chronic diseases of childhood, affecting more than 6 million children and more than 22 million persons in the United States [1]. The persistence or increase of asthma symptoms over time is accompanied by a progressive decline in lung functions [2]. The patho physiology of persistent asthma remains poorly understood. Even with the current therapies, the symptoms may be incompletely controlled, or they might have little effect on disease process [3, 4]. As pharmacologic therapy may have variable results in these asthmatic children, the search for metabolic or nutritional deficiencies contributing to the disease has continued. In instances where metabolic or nutritional deficiencies are a contributing factor to ongoing inflammatory processes in the bronchial tree, dietary supplementation of these deficiencies may provide benefit [5].

L-Carnitine is a cofactor that plays an essential role in the mitochondrial oxidation of long-chain fatty acids. It spares muscle glycogen, improves tolerance to physical activity, and reduces muscle fatigue [6]. It has been noted that L-Carnitine decreases leukotriene synthesis through inhibition of lipoxygenase enzyme [7]. L-Carnitine administration is beneficial to exercise and respiratory strength training in outpatients with stable, moderate-to-severe chronic obstructive pulmonary diseases [8]. L-Carnitine supplementation can increase serum carnitine levels, improve exercise capacity and correct the obstructive pattern in breath function tests in certain diseases [7, 9]. 

The role of L-Carnitine in development or persistence of pediatric asthma has not been investigated. This paper investigated the differences in serum L-Carnitine levels in pediatric asthmatic patients as compared to healthy control children. In addition, it studied the effectiveness of L-Carnitine supplementation in improving symptom scores and pulmonary function testing in children moderate persistent pediatric asthma. 

## 2. Study Patients

The study included 77 children between 6 and 12 years of age and diagnosed to have moderate persistent asthma. They were recruited from the Allergy and Pulmonology Unit, Paediatric Departments, New Children Hospital, Faculty of Medicine, Cairo University, Egypt. They were invited to participate in the study from March 2008 till May 2009. Fifty children out of 77 children completed the study, 10 refused to complete the study (as they were not able to swallow L-Carnitine capsules), and 17 failed to adhere to the study protocol (eight of them did not show up at the follow-up visits, 5 children did not take the asthma medications on a regular basis, and 4 suffered from infections during the pre study phase). Those 50 children were further randomly subdivided into two equal subgroups (A or B). Fifty healthy children of matched age and sex were included as a control group. 

## 3. Definition of Moderate Persistent Asthma

The children were defined as having moderate persistent asthma according to National Heart, Lung, and Blood Institute Guidelines, if they have daily symptoms, night-time awakenings more than 1 time/week but not nightly, some limitation of the normal activities, Forced Expiratory Volume in 1 second (FEV1) 60–80% of the predicted, Forced Expiratory Volume in 1 second/Forced Vital Capacity Ratio (FEV1/FVC) 75–80% of the predicted, daily use of a quick-relief inhaler, and/or exacerbations requiring systemic corticosteroids equal to or more than twice a year [1]. The standard care was allowed to be altered up or down during the supplementation phase according to the child condition.

## 4. Exclusion Criteria

1. Any pulmonary or chronic systemic diseases other than asthma and rhinitis, for example, cystic fibrosis, bronchiectasis, tuberculosis, diabetes mellitus, and liver.
2. Children with immunodeficiency or history of premature birth.
3. Children with hypothyroidism or borderline thyroid functions.
4. Other medications that may affect carnitine level: antibiotics use (particularly Ampicillins) and anticonvulsants for example Valproic acid.
5. Recent exhaustion, infection (especially pneumonia), surgery, anaesthesia.
6. Children who failed to have regular outpatient follow-up visits, a reliable caregiver, and willingness to complete at least 1 outpatient visit per month, and those who were not adherent to proper asthma medications.

## 5. Methodology

### 5.1. Study Design

There were two phases of the study; pre-clinical phase and active study phase during which children with moderate persistent asthma were recruited. During the 6-month preclinical phase the asthma control test, frequency of use of short acting B2 agonists, use of short courses of systemic steroids, the frequency of night time symptoms, frequency of acute asthma exacerbation, and the need for emergency department visit or hospitalization were recorded. During this phase every effort was done to maximize the asthma control and to decrease the severity of the asthma with the use of step up regimen according to National Heart, Lung, and Blood Institute Guidelines [1]. Children follow up and data collection were done at monthly intervals.

The active study phase was conducted as a randomized, double-blind, placebo-controlled trial over 6 consecutive months. The children were randomly allocated using computer-generated list of random numbers. At the beginning phase of this study, the total and free plasma carnitine levels were assessed in both the patient and control groups. One blood sample was collected from each of the control group, and two samples were collected from the asthmatic children; the first sample was obtained during acute asthma exacerbation, and the second was obtained three weeks after the attack. We considered the mean of the 2 samples for analysis. 

The patient group was further subdivided into two subgroups. Subgroup A (*n* = 25) received L-Carnitine 350 mg capsules, 3 capsules each morning for 6 months. Subgroup B (*n* = 25) received 3 placebo identical appearing capsules containing lactose for a similar time. The parents were strictly instructed to supervise their children while taking the capsules and to report the occurrence of diarrhoea or gastric upset as a sign of overdose. In addition, children were instructed to swallow and not to bite or chew the capsules. 

The children were checked at least once a month. They would also visit the clinic if they developed exacerbations or if it was necessary to maximize the asthma control. Recording of the childhood-asthma control test, the frequency of use of short acting B_2_ agonists, use of short courses of systemic steroids, the frequency of night time symptoms, frequency of acute asthma exacerbation, and the need for emergency department visit or hospitalization were also done as well as any side effects related to the medications including L-Carnitine. At the end of the study, the total and the free plasma carnitine levels were reassessed in the patient subgroups.

Serum total and free carnitine levels were studied by the enzymatic spectrophotometric method (Shimadzu UV160A spectrophotometer, Japan). A venous blood sample was withdrawn from each child on serum gel blood-collection tubes (Sarstedt). The obtained samples were centrifuged at 3000×g for 15 minutes, and serum samples were kept at −20°C until analysis. Serum total and free carnitine levels were measured by an enzymatic UV test kit (Roche Diagnostics GmbH, Mannheim, Germany, CAT. no. 11242008001). 

At the beginning of the study and at the end of treatment phase, the patients were assessed (using subjective, physiological & laboratory assessment) to avoid false positive or negative results as follows: *subjective *by childhood-asthma control Test (C-ACT) for children 4 to 12 years, *physiological* by pulmonary function test (PFT), and *laboratory* by sputum eosinophil and serum Immunoglobulin (Ig) E.

**Childhood-Asthma Control Test (C-ACT)**

The C-ACT is a 7-item child- and caregiver-completed tool with a scoring range of 0–27; higher scores indicate better control. A score of 19 or less indicates that the asthma may not be well controlled. The C-ACT is intended for use in children up to the age of 12 years [10].

**Pulmonary Function Tests (PFT)**

The children were weighed, and their heights were measured. Spirometry was performed for all children in the sitting position using a calibrated computerized spirometer (SpiroPro, CareFusion, San Diego, USA). Subjects were required to perform 3 acceptable Forced Vital Capacity (FVC) manoeuvres according to American Thoracic Society recommendations [1]. The maximum percentage in Forced Expiratory Volume in 1 second (FEV1) value from 3 readings was calculated.

The Kasr Al Eini Research Committee approved the study protocol. Verbal and written information were given with full explanation that the trial therapy is just an add-on therapy not replacing the traditional asthma therapy, and the children should continue their usual regimen of treatment. All the families were given the cell phone numbers of the investigators. They were instructed to call if there was any serious side effect of the L-Carnitine, any deterioration of the asthma condition or deterioration of the general condition of the enrolled child. A fully explained written consent was signed by the parents of the children who agreed to participate in the study. 

### 5.2. Statistical Analysis

The primary end point of this trial was improvement of the lung functions and C-ACT. We hypothesized that the use of L-Carnitine supplementations will significantly improve the lung functions and C-ACT and can reduce the severity of inflammation in children with moderate persistent asthma with *α* level being set to be 0.05. The power level of the primary end point of the study was more than 90% and was still more than 85% after excluding the 27 children who could not complete the study (using: *Power & Precision* V3; http://www.Power-Analysis.com/). We used the paired value and looked at the mean of the results of each treatment group. Data were presented as mean (±SD) values. The two-way analysis of variance (ANOVA) was used to identify statistically significant differences in the different parameters before and after the treatment. For all analysis, a statistical significance of *P* value <0.05 was used. The confidence intervals were calculated as indicators of standardized mean differences between groups. Wilcoxon's Signed Rank test was used to assess the normality of distributions of the data. Correlation between the free and total serum carnitine level and the severity of asthma was done. The statistical analysis was performed using TexaSoft, WINKS SDA Software, Sixth Edition, Cedar Hill, Texas, 2007.

## 6. Results

Demographic data of the children enrolled in the study were shown in Table 1. There were no important differences in the demographic features of those children who completed the study versus those who did not. There was no significant difference between patient and the control group as regard to age, BMI, and sex. The free and total carnitine serum levels (Table 2) were significantly lower in children with moderate persistent asthma than in the control group (*P* < 0.001). The side effects of L-Carnitine supplementation encountered during the study are shown in Table 3. None of these side effects enforced the children to stop the medication.  Table 4 demonstrates a significant positive correlation between both C-ACT and FEV1% predicted and the total and the free serum carnitine level. However, there was no significant correlation between these levels and the FEV1/FVC ratio with carnitine levels. 

Table 5 showed significant decrease in the emergency department visit, total hospital admissions, and blood eosinophils (%) between children supplemented with L-Carnitine compared to those asthmatic children with placebo or before supplementation, while there was no significant difference in serum IgE levels.  Table 6 showed no significant difference in free and total carnitine serum levels among patient subgroups before L-Carnitine supplementation (*P* > 0.05). However, this difference became significant after six months of oral carnitine supplementation (*P* > 0.001). The results of PFT and C-ACT before and after carnitine supplementation are shown in Table 6. There was no significant difference in PFT and C-ACT between patient subgroups (subgroup A & B) before L-Carnitine supplementation (*P* > 0.05). However this difference became significant after 6 months of L-Carnitine supplementation for both PFT and C-ACT (*P* < 0.001). Also there was a significant improvement of both PFT and C-ACT after L-Carnitine supplementation in the subgroup A children (*P* < 0.001). On the other hand, when comparing PFT and C-ACT in subgroup B before and after placebo supplementation, there was no significant improvement in FEV1% predicted and C-ACT. However, there was significant improvement in FEV1/FVC ratio.

## 7. Discussion

The increased asthma prevalence in the last few decades is due to different factors including changes in the dietary habits. Carnitine deficiency due to fad diets or lack of access in children may lead to different problems including failure to thrive, recurrent infections, hypotonia, encephalopathy, cardiomyopathy, or nonketotic hypoglycaemia [11]. Since L-Carnitine has an essential role in the transport of long-chain fatty acids through the mitochondrial membrane in order to ensure efficient *β*-oxidation of fatty acids, it is important for activation of pulmonary surfactant synthesis [12]. 

In the current study, there were significant lower free and total carnitine serum levels in children with moderate persistent asthma than in the controls. These findings agreed with the results of Asilsoy et al. who found that serum carnitine levels were decreased in children with moderate asthma during exacerbation of asthma and shortly thereafter [13]. Whether the decrease in serum carnitine in children with moderate persistent asthma is a cause or an effect, needs to be further studied. In the study conducted by Asilsoy et al., they attributed the decrease of serum carnitine levels during the asthmatic attacks to the decrease in lung surfactant (during attack) and the use of body stores to replenish it (after attack). 

Carnitine deficiency leads to toxic accumulation of long-chain fatty acids in the cytoplasm and of acyl CoA in the mitochondria. The accumulated saturated and monounsaturated fats may have different effects on airway inflammation [14]. Decreased serum carnitine was also documented in other respiratory problems like children with recurrent respiratory tract infections and neonates with respiratory distress syndrome [15, 16]. 

From our best knowledge, this study was the first to investigate the benefit of L-Carnitine supplementation in asthmatic children. In the current study, the PFT and the C-ACT were significantly improved in the asthmatic children who received L-Carnitine supplementation than in those who did not (*P* < 0.001). There was also a statistically significant improvement in PFT and C-ACT in subgroup A patients (who were supplemented with L-Carnitine) when compared to the presupplementation phase (*P* < 0.001). 

There are no previous data about the effect of L-Carnitine supplementation for asthma management in humans. In the animal model, Uzuner et al. studied the effect of L-Carnitine supplementation in mice with laboratory-induced asthma. They found that L-Carnitine supplementation improved oxygen saturation and decreased urine leukotriene E4 and inflammation in lung tissues in the studied animals [17]. 

The beneficial effect of L-Carnitine in asthmatic children may be due to different mechanisms. It may be due to its antimicrobial effect, muscle strength effects, enhancing surfactant synthesis, antileukotriene activity, or through antagonising the harmful effects of fat on the respiratory tract. Olgun et al. found an effective antimicrobial effect of L-Carnitine on different bacterial strains. They claimed that some of the side effects of orally administered L-Carnitine on gastrointestinal system like diarrhoea could probably be related to antimicrobial effect on local bacteria [18].

 Ergür et al. found that children with recurrent respiratory tract infections had low serum carnitine level [15]. Respiratory viral infections are important triggers of asthma attacks [19]. So children with low serum carnitine level may have recurrent viral respiratory tract infections and hence are more susceptible to develop asthma. Kavukçu et al. showed that carnitine can improve exercise tolerance and inspiratory muscle strength in COPD patients as well as reduce lactate production and increase rate of lactate removal [7]. 

Many studies proved that the surfactants of the asthmatic lungs are functionally impaired. The main mechanism of this impairment was thought to be due to the influx of inhibitory proteins into the airways, although changes in surfactant composition may occur [20]. Some studies proved the beneficial role of L-Carnitine supplementation in improving the synthesis and the functions of lung surfactants. Kurz et al., proved the beneficial role of L-Carnitine intake by pregnant women in decreasing the incidence of respiratory distress syndrome and even the mortality in premature newborns. They attributed these effects to the physiological action of L-Carnitine in surfactant synthesis activation [21]. 

Leukotrienes are among the mediators of inflammation in asthma and have a strong bronchoconstrictive effect. They are synthesized in the bronchial mucosa by eosinophils, basophils, and mast cells. They play an important role in airway eosinophilic inflammation, leukocyte trafficking, airway mucus secretion, airway oedema, collagen synthesis, and airway remodelling in asthmatic patients [22]. It has been reported that L-Carnitine inhibits leukotriene synthesis by inactivation of lipoxygenase pathway and by altering the ratio of essential fatty acids. Borghi-silva et al. and Ahmad et al. demonstrated that L-Carnitine was able to prevent bronchospasm and improve the obstructive findings in PFT in children undergoing chronic haemodialysis [8, 23].

The improvement of FEV1/FVC in the subgroup supplied with placebo treatment was of statistical significance but the improvement was very subtle regarding the figure. This may be due to the regular attendance at the clinic and closer followup and better asthma care during the study period or due to the few sample cases. Also, FEV1% predicted is much more important than FEV1/FVC in following the severity and the improvement of the lung function.

The limitation of the study is that the selection of patients was confined to children with moderate persistent asthma. We did not choose children with mild asthma as they can be easily controlled, and we did not choose children with severe asthma as they were too sick to be included in this kind of trial. Whether the study finding of carnitine effect can be applied to other degrees of asthma severity or other asthma subtypes or phenotypes needs to be further studied. 

Another limitation of this study is that we used a fixed dose of L-Carnitine. A dose effect of L-Carnitine supplement needs additional studies. An important limitation of the study is that despite the statistically significant improvement of C-ACT and PFT in the supplemented children, the children in the active treatment group still had unacceptably poor asthma control and were in need for more adequate asthma therapy. It is important to note that the L-Carnitine was investigated in the current study as add-on therapy and not replacing any of the traditional asthma medications. 

## 8. Conclusion

L-Carnitine levels were initially lower in moderate persistent asthmatic children as compared to healthy control children. Asthmatic children who received L-Carnitine supplementation showed significant statistical improvement of C-ACT and PFT. The target serum level of L-Carnitine, the effect of its supplementation in different phenotypes and degrees of asthma severity, and the appropriate dosing need more studies.

## Figures and Tables

**Table 1:** Demographic data and associated comorbidities in patients and control groups.

	Asthmatic (*n* = 50)	Control (*n* = 50)	*t*	*P*
Age	9 ± 2	9.14 ± 2	0.27	>0.05
BMI	19 ± 2	18.5 ± 1.9	1.43	>0.05
M/F	1.1 : 1	01 : 01.1		
Age at diagnosis	3 ± 0.90	—	—	—
Urban/rural	28 : 22 (1.27 : 1)	27 : 23 (1.17 : 1)		
Associated nasal allergy	27 (54%)	2 (4%)	—	<0.001*
Atopic dermatitis	17 (34%)	5 (10%)		<0.001*
Immediate family history of asthma	18 (36%)	1 (2%)	—	<0.001*
Smoking parents	1 (2%)	7 (14%)		

(*) *P* < 0.05 is significant.

There was no significant difference in age and BMI between asthmatic children and the controls while there was significant increase in the incidence of associated nasal allergy, atopic dermatitis, and family history of asthma in asthmatic children than in the control group. On the other hand the incidence of smoking parents was higher in control group than in the asthmatic group.

**Table 2:** Laboratory findings in patients and control groups before starting the L-Carnitine therapy.

	Asthmatic (*n* = 50)	Control (*n* = 50)	*t*	*P*
Serum IgE (kU/L)	325 ± 103	100.8 ± 60.8	13	<0.001*
Blood eosinophils (%)	5.9 ± 2.1	2.0 ± 0.9	12.5	<0.001*
Free carnitine (umol/L)	30.3 ± 1.8	39.3 ± 3.8	14.5	<0.001*
Total carnitine (umol/L)	40.1 ± 2.6	49.5 ± 3.9	12.8	<0.001*

(*) *P* < 0.05 is significant.

The table showed serum IgE, blood eosinophils %, free and total serum carnitine in patient and control groups before starting the L-Carnitine therapy. The serum IgE and blood eosinophils were significantly higher in asthmatic than in control children. On the other hand, the free and total serum carnitine levels were significantly lower in asthmatic than in control children.

**Table 3:** Side effects of L-Carnitine treatment.

GIT symptoms	2 (8%)
Stuffy nose	1 (4%)
Hyperactivity	1 (4%)
Headache	2 (8%)
Insomnia	0%
Palpitation	0%
Hypertension	0%

The table showed that, the most encountered side effects of L-Carnitine were the gastrointestinal disorders and headache. Blocked nose and hyperactivity; both were recorded in one child. Insomnia, palpitation or hypertension was not reported. None of these side effects enforced the patients to stop the intake of the oral L-Carnitine.

**Table 4:** Correlation between free and total carnitine levels with C-ACT, FEV1% of predicted and FEV1/FVC ratio in asthmatic children at the beginning of the study.

Variable (*n* = 50)		*r*	95% CI	*P*
C-ACT Mean ± SD 13.1 ± 1.43	Total S Carnitine 40.1±2.63	0.36	0.09, 0.58	<0.01*
	Free S Carnitine 30.3 ± 1.8	0.88	0.8, 0.9	<0.001*

FEV1 (% of predicted) 70.9 ± 3.7	Total S Carnitine	0.95	0.91, 0.97	<0.001*
	Free S Carnitine	0.7	0.52, 0.82	<0.001*

FEV1/FVC Mean ± SD 60.2 ± 4.5	Total S Carnitine	0.26	−0.02, 0.5	>0.05
	Free S Carnitine	0.07	−0.2, 0.3	>0.05
				

C-ACT: Childhood-Asthma control test. FEV1/FVC: Forced Expiratory Volume in 1st second/Forced Vital Capacity Ratio. (*) *P* < 0.05 is significant, *r* correlation coefficient, CI confidence interval.

The table showed the correlation between serum carnitine (free and total) and C-ACT and some pulmonary function parameters in asthmatic children at the beginning of the study. C-ACT and FEV_1_ (% of predicted) had significant positive correlations with total and free serum carnitine. However, there were no significant correlations between FEV_1_/FVC and the levels of total and free serum carnitine.

**Table 5:** Emergency room visit, total hospital admissions, blood eosinophils, and serum IgE in patient subgroups before and after carnitine supplementation and the placebo group.

		Subgroup A (with L-Carnitine) (*n* = 25)	Subgroup B (Placebo) (*n* = 25)	*t*	*P*
Emergency room visit	bef.	3.1 ± 1.01	3.4 ± 1.0	1.2	>0.05
	aft	1.5 ± 0.6	2.6 ± 0.9	4.68	<0.001*

Total Hospital admissions	bef.	1.5 ± 0.6	1.8 ± 0.7	1.9	>0.05
	aft	0.44 ± 0.5	1.8 ± 0.6	7.5	<0.001*

Oral steroid	bef.	2.16 ± 0.74	2.12 ± 0.72	0.21	>0.05
	aft	1.2 ± 0.5	1.6 ± 0.5	2.8	<0.01*

Serum IgE (kU/L)	bef.	328 ± 89	322 ± 114	0.21	>0.05
	aft	253 ± 62	290 ± 106	1.6	>0.05

Blood eosinophils (%)	bef.	5.8 ± 2.1	6.04 ± 2.1	0.38	>0.05
	aft	3.1 ± 0.8	4.4 ± 1.2	4.2	<0.001*

(*) *P* < 0.05 is significant There were significant decreases in the frequency of emergency room visit, total hospital admissions, and the intake of oral steroids between L-Carnitine-supplemented children compared to placebo group during the study period. The blood eosinophils % decreased significantly after treatment with L-Carnitine in asthmatic group. It was significantly lower in L-Carnitine treatment group than in placebo group. Serum Ig E showed no significant difference in treatment and placebo group (both before and after treatment).

**Table 6:** Free and total serum carnitine levels, pulmonary functions, and C-ACT in patient subgroups before and after carnitine supplementation and the placebo group.

		Subgroup A (with L-Carnitine) (*n* = 25)	Subgroup B (placebo) (*n* = 25)	*t*	*P*
Free carnitine (umol/L)	Bef.	30.3 ± 1.7	30.4 ± 1.8	0.17	>0.05
	Aft.	41 ± 5	31 ± 2	10	<0.001*

Total carnitine (umol/L)	Bef.	39.6 ± 2.3	40.6 ± 2.8	1.23	>0.05
	Aft.	50 ± 4	42 ± 3	47.56	<0.001*

FEV1% predicted	Bef.	70.5 ± 3.2	71.3 ± 4.2	*t*_1_ 0.66 *t*_2_ 15	>0.05 <0.001*
	Aft.	76.2 ± 3.4	71.4 ± 4.6	*t*_3_ 3.5 *t*_4_ 0.96	< 0.01* >0.05

FEV1/FVC	Bef.	60.1 ± 4.5	60.2 ± 4.6	*t*_1_ 0.8 *t*_2_ 25	>0.05 <0.001*
	Aft.	71 ± 4.4	61 ± 4.5	*t*_3_ 7.3 *t*_4_ 3.2	<0.001* <0.01*

C-ACT	Bef.	13.4 ± 1.5	12.8 ± 1.3	*t*_1_ 1.8 *t*_2_ 18	>0.05 <0.001*
	Aft	16.5 ± 1.7	13.3 ± 1.4	*t*_3_ 7.1 *t*_4_ 2.7	<0.001* >0.05


C-ACT: Childhood-Asthma control test. FEV1: Forced Expiratory Volume in 1 second. FEV1/FVC: Forced Expiratory Volume in 1st second/Forced Vital Capacity Ratio.

*t*_1_ between subgroup A & subgroup B before carnitine supplementation.

*t*_2_ between subgroup A Before & after 6 months of carnitine supplementation.

*t*_3_ between subgroup A & subgroup B after 6 months of carnitine supplementation to subgroup A.

*t*_4_ between subgroup B before & after 6 months of followup.

(*)* P* < 0.05 is significant.

There was significant increase in total and free serum carnitine levels in L-Carnitine-supplemented group than before starting the treatment and also when compared with the placebo group. After L-Carnitine supplementation, the pulmonary function tests and C-ACT showed significant improvement in the Carnitine supplemented group when compared to presupplementation or the placebo group. However, the total and free serum carnitine levels did not show any significant change after treatment in placebo group. There was also statistically significant improvement in pulmonary function tests and C-ACT in placebo group before and after treatment.
